# Accidental and Ambiguous Situations Reveal Specific Social Information Processing Biases and Deficits in Adolescents with Low Intellectual Level and Clinical Levels of Externalizing Behavior

**DOI:** 10.1007/s10802-020-00676-x

**Published:** 2020-08-13

**Authors:** M. M. Van Rest, M. Van Nieuwenhuijzen, J. B. Kupersmidt, A. Vriens, C. Schuengel, W. Matthys

**Affiliations:** 1 Faculty of Behavioural and Movement Sciences, Department of Clinical Child and Family Studies, Vrije Universiteit Amsterdam, Van der Boechorststraat 7, Amsterdam, 1081 BT the Netherlands; 2 Academic Collaborative Center ‘s Heeren Loo, Vrije Universiteit Amsterdam, Amsterdam, the Netherlands; 3 Research and Expertise Center William Schrikker, Amsterdam, the Netherlands; 4 Innovation Research & Training, Durham, North Carolina USA; 5 Stichting Leviaan, Department for Psychology, Psychiatry, and Adult Care, Purmerend, the Netherlands; 6 Department of Child and Adolescent Studies, Utrecht University, & Department of Psychiatry, University Medical Center Utrecht, Utrecht, the Netherlands

**Keywords:** Aggression, Social information processing, Social situations, Intellectual level, Externalizing behavior problems, Mild intellectual disability, Adolescent

## Abstract

Addressing aggression in youth requires understanding of the range of social problem situations that may lead to biased social information processing (SIP). The present study investigated situation-specificity of SIP and analyzed whether SIP deficits and biases are found in ambiguous as well as clearly accidental situations in adolescents with clinical levels of externalizing behavior or with low intellectual level, congruent with mild intellectual disability. Adolescents (*N* = 220, *M*_age_ = 15.21) completed a SIP test on a mobile app with six videos with ambiguous, hostile, and accidental social problems. Caretakers, teachers, and adolescents themselves reported on youth externalizing behavior problems. In accidental situations specifically, adolescents with low IQ scores more often attributed purposeful intent to perpetrators than peers with borderline or average IQ scores. In accidental situations, adolescents with clinical levels of externalizing behavior generated and selected more aggressive responses than nonclinical adolescents, regardless of their cognitive level. In line with previous literature, the ambiguous situations also brought out SIP differences between IQ groups. These results suggest that not only ambiguous situations should be considered informative for understanding SIP biases, but situations in which adolescents are clearly accidentally disadvantaged bring out SIP biases as well, that may lead to conflicts with others.

To better understand the social cognitive processes that underlie aggressive behavior in youth, the social information processing (SIP) model was developed by Dodge (1980) and adapted by Crick and Dodge (1994) and Lemerise and Arsenio (2000). This model asserts that in response to social problem situations, individuals respond with a sequence of mental operations, some of which may lead to aggressive behavior. In every social situation any person starts by encoding of internal and external cues, followed by the SIP step of making interpretations of the intent and emotions of self and others. Based upon these interpretations, the next SIP step contains the clarification of social goals, such as maintaining a good relationship or taking revenge. In the SIP step of response access a first spontaneous response is generated and several options of alternative responses are constructed. These responses could be prosocial and assertive, but they could also be passive or aggressive. Accordingly, in the SIP step including the decision process, different response options are evaluated as problem solutions, the self-efficacy for these responses is evaluated, and one response is selected. Finally, a behavioral response is enacted. While the associations between these SIP steps and aggressive behavior are now well-known, it is less well understood how biases in SIP are affected by the type of problem situation in which youth find themselves.

## Situation-Specific SIP

Research based on the SIP model shows that youth with aggressive and related externalizing behavior encode less information, are biased toward making hostile attributions of the intent of others in ambiguous situations, are prone toward generating aggressive response options, evaluate these responses positively, and select an aggressive response among several available responses (e.g., Fontaine et al. 2002; Oostermeijer et al. 2016; Orobio de Castro and van Dijk 2018; Raikes et al. 2013). In the seminal studies by Dodge and colleagues (Dodge 1980; Dodge and Frame 1982; Dodge et al. 1984) - where SIP was examined in situations varying in intent by the perpetrator - ambiguous situations were shown to differentiate in hostile intent attribution between children with and without externalizing behavior. The successive research agenda primarily focused on understanding children’s SIP in social situations where the intent of a perpetrator was ambiguous (see meta-analysis by Orobio de Castro et al. 2002). It is therefore unknown to what extent SIP biases are activated in other situations where youth are clearly accidentally disadvantaged. Because SIP among children with clinical levels of externalizing behavior (from this point on described as “behavior problems”) may depend upon the type and intensity of the problem situation, such as being disadvantaged and coping with competition or provocation (Helseth et al. 2015; Matthys et al. 1999), testing SIP as a function of situations that vary in perpetrator intent was the first main goal of the current study.

## Accidental Problem Situations for Youth with Low Intellectual Level and Behavior Problems

Situation-specific SIP is particularly relevant for youth with a low intellectual level, congruent with mild intellectual disability (ID; IQ ≤ 70 with adaptive functioning problems) and borderline intellectual functioning (71–84; Schalock et al. 2010) as these youth with ID and borderline intellectual functioning have a high risk for aggression and other externalizing behaviors (Douma et al. 2007) and are overrepresented in the youth criminal justice system (Kaal 2010). Indeed, these youth more often misinterpret social cues from ambiguous situations in a hostile manner (e.g., Van Nieuwenhuijzen et al. 2011), they encode less cues and with limited information available to inform decision-making in social situations they are more likely to make decisions that result into negative interactions with adults and peers (Van Nieuwenhuijzen et al. 2004; Van Nieuwenhuijzen et al. 2009a; Van Nieuwenhuijzen et al. 2011). Youth with ID and borderline intellectual functioning may not only show SIP difficulties in ambiguous (or clearly hostile) situations, but also in situations when they are clearly accidentally disadvantaged. They may become confused when there is a benign intent on the part of a perpetrator, but a negative outcome for a victim (e.g., Gomez and Hazeldine 1996). Attributing an accidental or benign nature to a perpetrator’s intent when there is a clear negative outcome of the situation requires that not only the damage to the victim is processed, but the apparent intention of the perpetrator and the lack of preceding provocation as well; it may be a more complex process than the attribution of hostile intent and it may produce more cognitive load while processing (Rosset and Rottman 2014). These positive and negative aspects of the situation have to be integrated into one conclusion. Particularly for youth with ID, this integration could be too difficult, especially when fast processing of the social problem is required (Rosset 2008). In fact, Leffert and Siperstein (1996) examined the mechanisms of encoding and intent attribution in younger children with intellectual disability in benign or accidental situations. They found that these children accurately interpreted hostile intentions, but had difficulty in accurately interpreting benign intentions (Leffert and Siperstein 1996), and were less often correct in intent attributions compared to typically developing peers (Leffert et al. 2010).

Building on the seminal studies by Dodge and colleagues (Dodge 1980; Dodge and Frame 1982; Dodge et al. 1984) on child intent attributions in various situations, it is also expected that accidental situations may provide complex processing for adolescents with behavior problems. These adolescents more often have negative cognitive schemas (e.g., mistrusting others) which have a negative influence on SIP skills (Calvete and Orue 2012). Within accidental situations these schemas may influence SIP when there is a negative outcome with an incongruent benign intent. Investigating this situation-specificity for SIP skills in adolescents with low intellectual level, congruent with mild ID, and in adolescents with behavior problems was our second main goal of the study.

## Full Scope of SIP Skills in Adolescents

Following Leffert and Siperstein’s (1996) work on children’s interpretation skills in accidental situations, the current study added as a third goal to examine not only SIP steps from the early phases in different situations, but also the middle and late phases of the SIP model. Combining our three main goals, this study aimed to test SIP as a function of situations that vary in perpetrator intent, and to investigate this situation-specificity for multiple SIP skills from the early, middle and late phases in adolescents with low intellectual level, congruent with mild ID, and in adolescents with behavior problems.

This study hypothesized that accidental situations, as well as previously studied ambiguous situations, would not only elicit group differences on the early SIP skills, but also on the middle and later SIP skills of generation, self-efficacy, positive evaluation and selection of aggressive responses and related feelings of anger. As youth with ID have difficulties with cognitive load and integrating benign features of situations and show impaired impulse control (Leffert and Siperstein 1996; Rosset and Rottman 2014; Van Nieuwenhuijzen et al. 2009b), and because youth with behavior problems often have biased cognitive schemas, these adolescents likely have difficulty suppressing or regulating their feelings of anger and inhibiting aggressive responses in accidental situations. Because youth with ID and with behavior problems are less likely to interpret benign cues, they are more inclined to interpret social situations with a hostile intent. As a result, they are hypothesized to be more prone to positively evaluate, have self-efficacy for, and select an aggressive response as a reaction to that situation, even if the situations were accidental or ambiguous in nature. For the SIP skill of encoding cues in adolescents with ID, differences were not hypothesized for accidental or ambiguous situations specifically, as this skill is a more neutral cognitive skill in a social situation. Because adolescents with ID have a general cognitive deficit, impairments in encoding were expected to be more pervasive for adolescents with ID, and less situation-specific.

## Method

### Participants

There were 233 13 to 17-year-olds (*M* = 15.22 years old, *SD* = 1.34; 46% female) recruited to participate in the study. A total of 13 adolescents were removed from the analyses due to either missing data on one of the main variables of this study or due to an IQ-estimate score below 50, resulting in a final sample of 220 participants (*M* = 15.21 years old, *SD* = 1.35; 46% female). Adolescents came from across the entire country, including urban and rural areas. To achieve a stratified design including a representative sample of youth within a range of externalizing behavior and intellectual ability, youth were invited from residential clinical care institutions and special residential care institutions for youth with mild ID or borderline functioning (*n =* 57; 26%), youth psychiatric (day) care institutions (*n =* 51; 23%), special education programs (*n =* 76; 35%), and mainstream education for a control group with average intelligence (*n =* 36; 16%). Youth from institutions and special education programs routinely received clinical assessment as part of their enrollment; only those youth diagnosed with mild ID, borderline intellectual functioning, and/or (clinical) behavior problems were placed in these settings from which we recruited our sample.

The participants were divided into three groups, based upon their estimated intelligence scores such that 91 participants (41%) were classified as having a Low IQ score (LIQ), congruent with the intellectual level of a mild intellectual disability (IQ 50–70; see Schalock et al. 2010; and DSM-5), 61 participants (28%) had a Borderline IQ score (BIQ; 71–84) referring to the borderline between average level and mild intellectual disability, and 68 participants (31%) had an Average IQ score (AIQ ≥ 85). To investigate SIP deficits and biases related to either intellectual level or behavioral functioning, each IQ group was divided into two groups based upon their behavior problem scores (see under Measures below), resulting in 115 adolescents (52%) with clinical levels of externalizing behavior and 105 adolescents (48%) in the normal range of behavior scores. Table 1 shows the six groups of participants defined by IQ and behavior problems.

**Table 1:** Demographics of participant groups based on Cognitive Level and Externalizing Behavior differences

	LIQ-EXT *n* = 55		LIQ-NON *n* = 36		BIQ-EXT *n* = 35		BIQ-NON *n* = 26		AIQ-EXT *n* = 25		AIQ-NON *n* = 43		IQ*EXT		
	*M*	*SD*	*M*	*SD*	*M*	*SD*	*M*	*SD*	*M*	*SD*	*M*	*SD*	*F (or X*^*2*^*)*	*p*	*n*^*2*^
IQ-est	61.73^a^	5.76	62.69^a^	6.41	76.74^b^	3.12	77.23^b^	3.99	92.44^c^	8.84	101.19^d^	12.11	6.28	.00	.06
Ext. T-score	70.02^a^	4.52	54.78^b^	6.02	72.26^a^	7.39	54.42^b^	5.99	71.92^a^	5.52	54.28^b^	5.73	1.18	ns	.01
Age	15.32	1.26	15.35	1.39	15.12	1.30	15.05	1.58	15.12	1.53	15.18	1.27	.04	ns	.00
SES	2.80^a^	1.54	3.94^bc^	1.17	3.19^ac^	1.17	3.74^bc^	.96	4.07^bc^	1.27	4.63^b^	1.45	1.26	ns	.01
% Male*	54.50		58.30		51.40		57.70		64.00		44.20		3.19	ns	
% Min stat*	29.10		11.10		22.90		19.20		44.00		20.90		10.09	ns	

Note. Means with different letter superscripts are significantly different from one another, based on interaction or main effect

LIQ = low IQ score. BIQ = borderline IQ score. AIQ = average IQ score

EXT = clinical levels of externalizing behavior. NON = non-clinical behaviors

IQ*EXT = the interaction effect between cognitive level and externalizing behavior

IQ-est = full-scale IQ estimated score from Wechsler intelligence test

Ext. T-score = externalizing behavior problems highest T-score out of CBCL, TRF, or YSR

SES = socioeconomic status indicator. Min stat = race-ethnicity minority status

*Percentages per group and tested by Pearson Chi-square

### Measures

#### Demographics

Information about participants’ age, gender, race- ethnicity minority status, and socioeconomic status (SES) was obtained from a questionnaire completed by the parents or the legal guardian of the adolescent. Minority status was defined as a combination score based upon the birth country of both parents and the adolescent, as is the official minority registration in the Netherlands (Simon 2012). In the Netherlands , the four most common and official minority groups are from Turkey, Morocco, Surinam, and the Dutch Antilles (Central Bureau of Statistics 2020; sample percentages of 4.5%, 4.1%, 3.2%, and 2.3%, respectively, indicated slight overrepresentation compared to population percentages). Other birth countries of small minority groups in the Netherlands were also included in the minority status category (for example there were *n* = 3 from Asian countries, *n* = 6 from African countries), resulting in a dichotomous score for either belonging to a minority group or not. SES approximation was defined by the maximum score of the highest level of education of both parents, answered on a scale ranging from 0 for no formal education to 7 for post-doctoral education.

#### Cognitive Level

Cognitive level was assessed using the subtests “Vocabulary” and “Block Design” of the Wechsler intelligence tests (Wechsler 1949, 1955). The Dutch version of the WISC-III was used for all participants under 17 years of age (Kort et al. 2005) and the WAIS-III/IV (Uterwijk 2000) was used for participants who were 17 years old (*n* = 23). Cognitive level was estimated using a global intelligence formula for approximation of Full-Scale IQ (FIQ) with the sum of the scaled subtest scores of the subtests “Vocabulary” and “Block Design” (e.g., Hrabok et al. 2014; Silverstein 1970). After calculation of the FIQ-estimate, the sample was divided into three IQ groups including Low IQ (LIQ), congruent with the intellectual level of a mild intellectual disability (IQ 50–70), Borderline IQ (BIQ 71–84), and Average IQ (AIQ ≥85). Because in following analyses the wider confidence intervals of IQ-estimates could not be included in this variable, we decided not to use the term ‘mild to borderline intellectual disability’ for the groups we created for our analyses; therefore, in all following sections of method and results these groups are named after merely IQ-score: LIQ, BIQ, and AIQ. (Information about number of participants with FIQ scores from the year previous to our study and missing data for all following variables can be obtained from the first author).

#### Externalizing Behavior

Externalizing behavior was assessed using the ASEBA scales (Achenbach 2009): Child Behavior Checklist (CBCL/6–18); Teacher Report Form (TRF/6–18) and Youth Self Report (YSR/11–18). The syndrome scales “Aggression” and “Rule breaking behavior” were combined into a total Externalizing Behavior Problem scale. Teachers (TRF), caretakers (CBCL), and adolescents (YSR) completed the 32, 35, and 32 items, respectively, of these two syndrome scales of the Dutch ASEBA versions. Each item on the Externalizing behavior scale describes aggressive or rule breaking behaviors rated on a 3-point Likert scale: 0) not true, 1) sometimes or somewhat true, or 2) often or very true. The dependent variables were the total raw scores for Externalizing behavior on all three questionnaires with their corresponding T-scores, calculated separately for boys and girls. A composite score was created from these variables by taking the highest T-score of either the TRF, CBCL, or YSR reports, maximizing the sensitivity of this variable, based on procedures described by Angold (2002) and Bird et al. (1992). Subsequently, a dichotomous score for behavior problems was used to distinguish and assign youth to one of two groups, either below the 98th percentile (non-clinical level) and at or above the 98th percentile (clinical level) based on their highest Externalizing behavior T-score (Achenbach 2009).

#### Social Information Processing

Social information processing was assessed through adolescents’ responses to six videos depicting a range of social problem situations. Participants responded to both open-ended and multiple-choice questions on a tablet computer in a mobile SIP app called SIVT (Van Rest et al. 2019).

Three types of interpersonal problem situations showed something negative or bad happening to a victim. In two videos, the intent of the perpetrator was designed to be clearly hostile; in two videos the intent was ambiguous; and in two videos the intent was clearly accidental or benign. Within each type of situation (i.e., hostile, ambiguous, accidental), one video involved a peer perpetrator and another involved an adult perpetrator. The plots for these videos were chosen based upon interviews with adolescents about the social situations which they found to be most difficult or challenging (Van Bokhoven et al. 2011). The content, ecological, face, and criterion validity of the instrument were established in two studies including clinical samples and a norm population study. Reliability measures are presented for particular variables below (see full psychometric properties by Van Rest et al. 2018, 2020).

Encoding was assessed by responses to the open-ended question “What happened in this video?” The 10 most essential cues in the social situation were scored from the verbal answer by the respondent. One point was awarded per cue mentioned, leading to scores ranging from 0 to 10 correct per video. Across the six videos of the current study, the overall internal consistency of the measure was good (α = .85) and would decrease if any single item was deleted. Inter-rater reliability kappas for encoding items were calculated for each of the six videos separately; for video A, _K_ = .87; B, _K_ = .66; C, _K_ = .67; D, _K_ = .62; E, _K_ = .77; F. _K_ = .59. Mean scores were calculated across six videos and for each of the three situation types, namely, hostile, ambiguous, and accidental. This accounted for all following variables.

Interpretation was assessed by administering two multiple-choice items for each video. The first item examined attributions of the perpetrator’s Hostile Intent by asking “What do you think of the intent of [this boy/girl/parent/teacher]?” Answers were given on a three-point Likert-type scale, 1 = normal/neutral, 2 = a little mean, 3 = (very) mean. The second item examined Purposeful Intent using the question, “X happened, [this perpetrator] did that…” Response options for these items were 1 = definitely by accident, 2 = perhaps/probably by accident, 3 = perhaps/probably on purpose, 4 = definitely on purpose. For the interpretation cognitive mechanism and subsequent generation of responses, feelings of anger, evaluation, and selection mechanisms, Cronbach’s alphas were not calculated, as these variables were hypothesized to interact with the situations presented.

Aggressive response generation was assessed with the open-ended question: “If this happened to you, what would you do?” Answers were dichotomized into either an aggressive or antisocial response versus a passive, assertive, or prosocial response. For this SIP skill, sum scores were calculated instead of mean scores. Inter-rater reliability kappas for this open-ended item for each of the six videos separately were; for video A, _K_ = .41; B, _K_ = .71; C, _K_ = .59; D, _K_ = .76; E, _K_ = .60; F. _K_ = .70.

Anger was assessed by: “How would you feel?” Participants answered on a five-point Likert-type scale ranging from 1 = calm to 5 = furious.

Evaluation and Self-efficacy for aggressive responses were measured after randomly presenting three videos with an aggressive, assertive, or passive response as possible reaction by the victims in each of the six situations. Multiple-choice questions were posed to assess the participant’s self-efficacy for behaving aggressively (“Could you also respond like [this victim]?”), and to assess aggressive response evaluation skills (“Will things work out, if [the victim] did that?”). The participants answered on a 5-point Likert-type scale, ranging from 1 = totally not, to 5 = totally.

Aggressive response selection was assessed by presenting pictures of the three response videos – aggressive, assertive, and passive – while asking: “Which one would you choose?” One point was scored each time a participant selected the picture of an aggressive response as his or her response to handle the situation. Sum scores for aggressive response selection were calculated in total and for each situation type.

### Procedure

The current study was approved in 2013 by the Science and Ethics Committee of the Faculty of Behavioural and Movement Sciences and by the Ethical Committee of the Institute for Health and Care Research at the medical center of the Vrije Universiteit Amsterdam . Clinical professionals from several institutions and schools, and Master’s students were trained on the administration of a standardized data collection protocol. After the parent or legal guardian provided parent permission and informed consent, the adolescent provided written assent to participate in the study, and boards provided permission for institutions or schools to participate in the study, assessments were conducted at clinical institutions and schools. Assessments were individually administered using a semi-structured protocol and had a mean duration of 80 min. A test administrator presented all questions verbally. In addition, adolescents could read along on the tablet computer which provided them with visual support. Adolescents received a small monetary incentive for their participation.

## Results

### Overview of the Analyses

To investigate situation-specific SIP skills from the early, middle, and late phases for adolescents with LIQ and with behavior problems the main analyses involved one repeated measures MANCOVA with a factorial design including 2 between subjects factors, namely, Cognitive Level (LIQ, BIQ, AIQ) and Externalizing Behavior (clinical range, non-clinical range), and 1 within subjects factor, namely, Situation Type (hostile, ambiguous, and accidental intent) across eight SIP skills as outcomes including the two covariates SES and Minority Status.

First, to answer whether SIP in general is situation-specific the multivariate main effect of Situation Type was reported for overall SIP differences. Second, to answer whether SIP is situation-specific for adolescents with LIQ and adolescents with behavior problems the multivariate two-way interaction effects between Situation Type and either Cognitive Level or Externalizing Behavior were examined. Main effects were also presented for overall SIP differences between IQ groups or between behavior groups, given the possibility of non-significant interaction effects.

Third, to test our hypotheses about situation-specificity for LIQ or behavior problem groups of each SIP skill separately, the univariate results from the MANCOVA were analyzed. Situation-specific and pervasive differences between groups were examined for each of the eight SIP skills by AN(C)OVAs, showing interaction effects, main effects, and using post hoc probing tests. Univariate main effects of Situation Type, Cognitive Level, and Externalizing Behavior were interpreted per SIP skill in case of non-significant interaction effects. Cohen’s criteria were used for interpreting effect sizes.

### Demographic Statistics

Mean IQ and Externalizing behavior problem scores differentiated well between the study subgroups (See Table 1 for specific information). All six groups were comparable in their age and gender composition. Given the skew in distribution of SES and race-ethnicity minority status between some groups, these variables were included as covariates in subsequent analyses (see Table 1).

### Situation-Specific SIP Bias in General

Multivariate main effects analysis of the within subjects factor of Situation Type showed that specific situations triggered more overall SIP biases and deficits within all adolescents than other situations. There was a significant and strong multivariate main effect for the within subjects factor of Situation Type (Pillai’s Trace *V* = .47, *F*(16,197) = 10.93, *p* < .001, *η*^2^ = .47).

### Situation-Specific SIP Bias for Adolescents with Low Intellectual Level or Behavior Problems

Multivariate two-way interactions from the overall MANCOVA showed that overall SIP differences between situations were significantly different for LIQ, BIQ, and AIQ groups, based on the strong multivariate two-way interaction for Situation Type by Cognitive Level (Pillai’s Trace *V* = .29, *F*(32,396) = 2.11, *p* = .001, *η*^2^ = .15). The SIP differences between situations specified for the two behavior groups were also statistically significant and strong, indicated by the multivariate two-way interaction for Situation Type by Externalizing Behavior (Pillai’s Trace *V* = .14, *F*(16,197) = 1.95, *p* = .02, *η*^2^ = .14). Thus, adolescents with lower cognitive level or higher externalizing behavior level showed more overall SIP deficits and biases in certain situations specifically.

Because the multivariate interaction effect between Cognitive Level and Externalizing behavior was not significant (Pillai’s Trace *V* = .08, *F*(16,412) = 1.03, *p* = .43, *η*^2^ = .04), the multivariate main effects were also reported. A significant and strong multivariate main effect was found for Cognitive Level (Pillai’s Trace *V* = .22, *F*(16,412) = 3.11, *p* < .001, *η*^2^ = .11), with significant univariate effects on six SIP skills (see paragraphs below). A significant and strong multivariate main effect was found for the Externalizing Behavior factor (Pillai’s Trace *V* = .14, *F*(8,205) = 4.01, *p* < .001, *η*^2^ = .14), with significant univariate effects for three SIP skills (see paragraphs below). Thus, adolescents with lower cognitive level and adolescents with higher externalizing behavior level showed pervasive overall SIP deficits or biases when all situations were taken together.

### Understanding Situation-Specific and Pervasive Group Differences for each SIP Skill Separately

The univariate interaction effects and main effects from the overall MANCOVA were examined per SIP skill separately. There were six SIP skills with significant interactions from the MANCOVA (see Table 2). Subsequent repeated measures AN(C)OVAs were performed to probe these interaction effects for each of the SIP skills and to describe the specific differences by post hoc tests.

**Table 2:** The significant univariate interaction effects for six SIP skills from the overall repeated measures MANCOVA

	*SS*	*df*	*MS*	*F*	*p*	*η*^2^
Purposeful Intent on Situation by Cognitive Level	3.87	4424	.97	2.78	.03	.03
Anger on Situation by Cognitive Level	5.90	4424	1.48	4.95	.00	.05
Evaluation of Agg on Situation by Cognitive Level	4.41	4424	1.10	3.74	.01	.03
Selection of Agg on Situation by Cognitive Level	1.71	4424	.43	2.63	.03	.02
Generation of Agg on Situation by Externalizing Behavior	3.49	2424	1.75	6.89	.00	.03
Selection of Agg on Situation by Externalizing Behavior	1.02	2424	.51	3.12	.045	.01

*Agg* Aggressive Responses

**Fig. 1:**
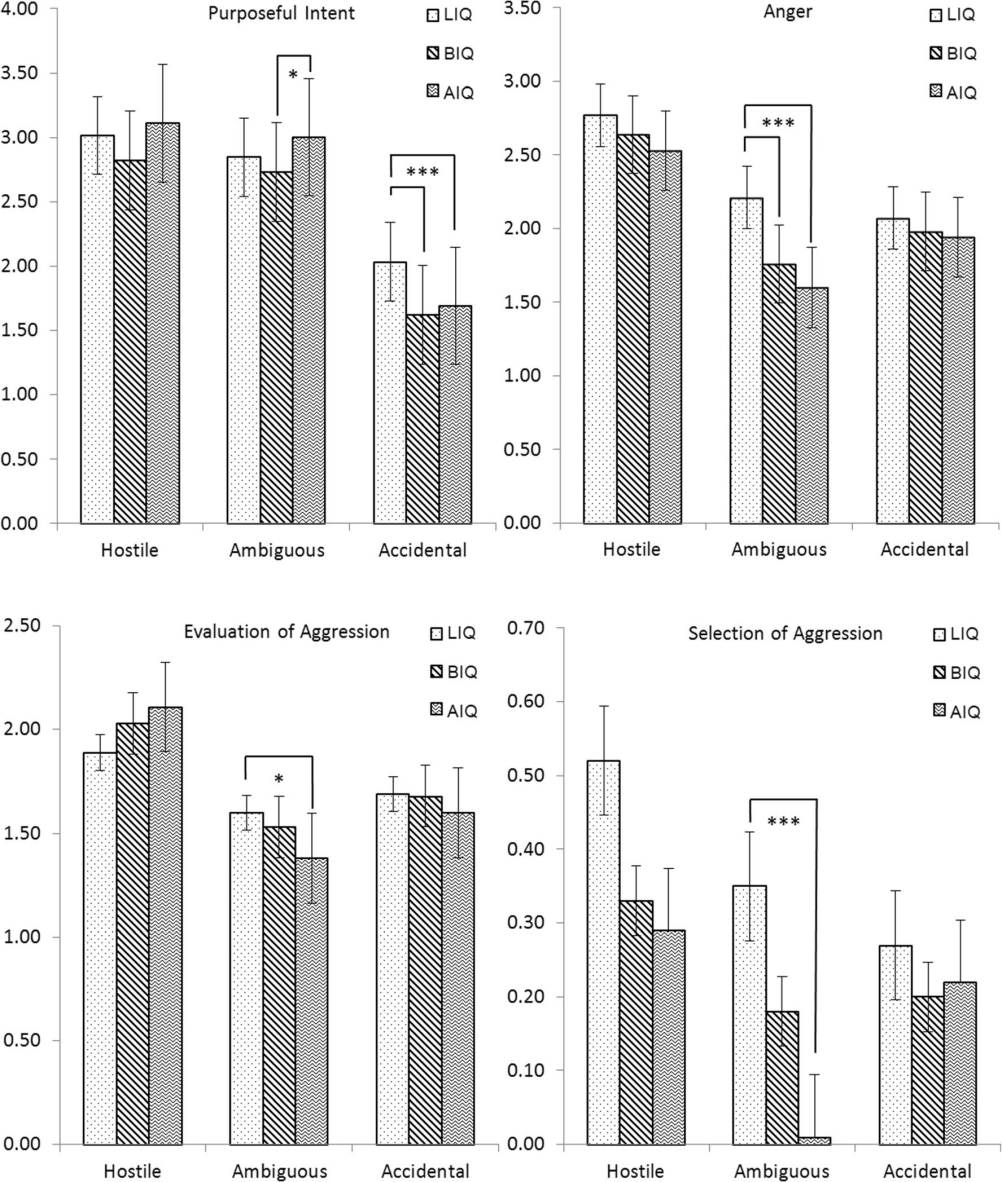
Two-way interaction effects of Situation Type by Cognitive Level in four different SIP skills. Note. Significant post-hoc group differences within situations are indicated by * *p* < .05, ** *p* < .01. *** *p* < .001. LIQ = low IQ score. BIQ = borderline IQ score. AIQ = average IQ score

**Fig. 2:**
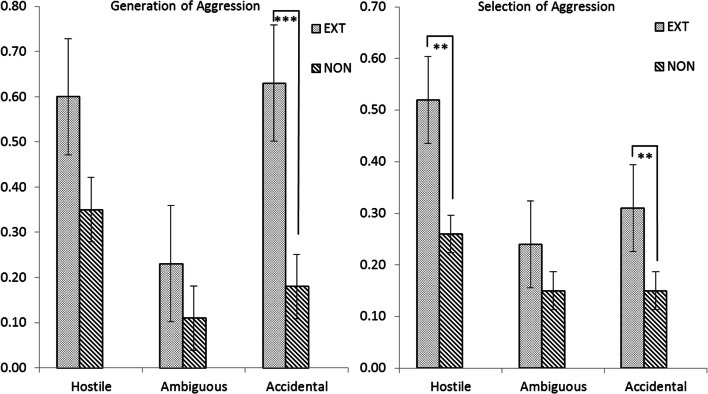
Two-way interaction effects of Situation Type by Externalizing Behavior in two different SIP skills. Note. Significant post-hoc group differences within situations are indicated by * *p* < .05, ** *p* < .01. *** *p* < .001. EXT = clinical levels of externalizing behavior. NON = non-clinical behaviors

The univariate ANCOVAs confirmed four significant two-way interactions between Situation Type and Cognitive Level (Fig. 1) and two significant two-way interactions between Situation Type and Externalizing Behavior (Fig. 2). For other SIP skills, if two-way interactions were not significant, the main effects were examined. For these pervasive differences per SIP skill, means, standard deviations, and F test results from main effects analyses can be seen in Table 3 for Situation Type, in Table 4 for Cognitive Level, and in Table 5 for Externalizing Behavior.

**Table 3:** Within subject main effects of Situation Type on separate SIP skills

	Hostile		Ambiguous		Accidental				
	*M*	*SD*	*M*	*SD*	*M*	*SD*	*F*_2,218_	*p*	*η*^2^
Encoding	4.44^a^	1.20	5.51^b^	1.41	4.86^c^	1.34	113.92	.00	.51
Hostile Intent	2.19^a^	.53	1.61^b^	.47	1.35^c^	.40	268.11	.00	.71
Purposeful Intent*	2.99^a^	.71	2.86^a^	.64	1.81^b^	.66	221.12	.00	.67
Generation Agg*	.48^a^	.69	.18^b^	.39	.41^a^	.65	25.46	.00	.19
Anger*	2.66^a^	.81	1.90^b^	.75	2.00^b^	.61	106.71	.00	.50
Self-Efficacy Agg	2.88^a^	1.20	2.31^b^	1.14	2.69^c^	1.03	33.31	.00	.23
Evaluation Agg*	2.00^a^	.86	1.51^b^	.63	1.66^c^	.66	42.04	.00	.28
Selection Agg*	.40^a^	.65	.20^b^	.49	.24^b^	.50	12.61	.00	.10

Means with different letter superscripts are significantly different from one another

*Significant interactions with Externalizing Behavior or Cognitive Level were found for these SIP skills

Agg = Aggressive Responses

**Table 4:** Between subjects main effects of Cognitive Level with SES as covariate and SIP skills as the dependent variables

	LIQ *n* = 91		BIQ *n* = 61		AIQ *n* = 68				
	*M*	*SD*	*M*	*SD*	*M*	*SD*	*F*_2,216_	*p*	*η*^2^
Encoding	4.53^a^	1.03	4.93^ab^	1.20	5.48^b^	1.09	9.06	.00	.08
Hostile Intent	1.84^a^	.37	1.66^b^	.30	1.59^b^	.35	8.13	.00	.07
Purposeful Intent*	2.63^a^	.51	2.39^b^	.41	2.60^a^	.37	5.94	.00	.05
Generation Agg	1.27	1.40	.93	1.08	.93	1.20	1.21	ns	.01
Anger*	2.35^a^	.65	2.13^ab^	.50	2.02^b^	.44	5.83	.00	.05
Self-Efficacy Agg	2.85^a^	.90	2.63^ab^	.99	2.32^b^	.90	3.80	.02	.03
Evaluation Agg*	1.73	.58	1.75	.63	1.69	.49	.36	ns	.00
Selection Agg*	1.14^a^	1.59	.70^ab^	1.16	.53^b^	.89	5.20	.01	.05

Means with different letter superscripts are significantly different from one another

*Significant interactions with Situation Type were found for these SIP skills

Agg = Aggressive Responses. LIQ = low IQ score. BIQ = borderline IQ score. AIQ = average IQ score

**Table 5:** Between subjects main effects of Externalizing Behavior with SES and Minority Status as covariates and SIP skills as the dependent variables

	EXT *n* = 115		NON *n* = 105				
	*M*	*SD*	*M*	*SD*	*F*_1,216_	*p*	*η*^2^
Encoding	4.73	1.19	5.15	1.10	1.87	ns	.01
Hostile Intent	1.76	.36	1.66	.36	1.74	ns	.01
Purposeful Intent	2.58	.44	2.53	.46	.77	ns	.00
Generation Agg*	1.46	1.32	.65	1.05	16.06	.00	.07
Anger	2.26	.57	2.11	.60	1.64	ns	.01
Self-Efficacy Agg	2.95	.95	2.27	.82	25.72	.00	.11
Evaluation Agg	1.77	.60	1.68	.53	1.97	ns	.01
Selection Agg*	1.08	1.45	.56	1.09	9.80	.00	.04

Note. * Significant interactions with Situation Type were found for these SIP skills

Agg = Aggressive Responses

EXT = clinical levels of externalizing behavior. NON = non-clinical behaviors

#### Encoding

No significant interaction effects were found for Encoding; however, a pervasive difference was found between situation types. When the intent of the perpetrator was clearly hostile, adolescents significantly encoded the smallest number of cues, they encoded more cues in accidental situations, and most cues were encoded when the situation was ambiguous. A main effect for Cognitive Level was also found such that adolescents with LIQ encoded fewer cues than AIQ adolescents. The LIQ and AIQ groups did not differ significantly from the BIQ group. These main effect analyses revealed that the SIP skill of Encoding was pervasively impaired in adolescents with LIQ across all situations. No significant effects were found for Externalizing Behavior.

#### Interpretation of Hostile Intent

A significant main effect for Situation Type showed all groups to interpret most hostile intent in videos where indeed the intent of the perpetrator was clearly hostile, compared to lower interpretation in ambiguous or accidental situations. In addition, a significant main effect for Cognitive Level revealed that adolescents in the LIQ group were significantly more likely to have a hostile attributional bias across all situations than adolescents in the BIQ and AIQ groups, who did not differ significantly from one another. The SIP skill of Interpretation of Hostile Intent was pervasively elevated in adolescents with LIQ in all types of situations. No significant main effect was found for Externalizing Behavior.

#### Interpretation of Purposeful Intent

A significant two-way interaction of Situation Type by Cognitive Level was found for Purposeful Intent (Pillai’s Trace *V* = .05, *F*(4,424) = 2.69, *p* = .03, *η*^2^ = .03) with no effects for the covariates. Post-hoc probing showed that specifically in situations where the intent of the perpetrator was accidental, adolescents with an LIQ were significantly more likely to believe that the perpetrator acted on purpose (*M* = 2.03, *SD* = .73) than youth in the BIQ (*M* = 1.62, *SD* = .51) and AIQ groups (*M* = 1.69, *SD* = .61; *F*(2,216) = 8.63, *p* < .001, *η*^2^ = .07), who did not differ significantly from one another (see Fig. 1). The mean scores indicated that adolescents with BIQ and AIQ were more certain about the accidental or benign nature of the perpetrator’s intent than adolescents with LIQ. In the ambiguous situations, the BIQ group scored somewhat lower on Purposeful Intent (*M* = 2.73, *SD* = .64) than the AIQ group (*M* = 3.00, *SD* = .53; *F*(2,216) = 3.25, *p* = .04, *η*^2^ = .03). These mean scores suggested that the adolescents with BIQ, on average, were less certain about whether the perpetrator meant to do it on purpose or not and the AIQ group, including adolescents with and without behavior problems, was more inclined to think it may be a bit on purpose. In these ambiguous situations, neither group differed significantly from the LIQ group (*M* = 2.85, *SD* = .69), and in clearly hostile situations, no significant differences for Purposeful Intent were observed between the groups. No effects were found for Externalizing Behavior.

#### Generation of Aggressive Responses

A significant two-way interaction of Situation Type by Externalizing Behavior was found for Generation of Aggressive Responses (Pillai’s Trace *V* = .07, *F*(2,211) = 8.15, *p* < .001, *η*^2^ = .07), without effects for the covariates. Post-hoc probing showed that specifically in situations with accidental perpetrator intent, adolescents with behavior problems significantly generated more aggressive responses (*M* = .63, *SD* = .73) than non-clinical adolescents (*M* = .18, *SD* = .43; *F*(1,216) = 24.14, *p* < .001, *η*^2^ = .10; see Fig. 2). This difference between behavior problem groups was not found for ambiguous or hostile situations. No effects were found for Cognitive Level.

#### Anger

A significant two-way interaction of Situation Type by Cognitive Level was found for Anger (Pillai’s Trace *V* = .09, *F*(4,424) = 5.24, *p* < .001, *η*^2^ = .05), without effects for the covariates. Post-hoc probing tests strongly showed that in ambiguous situations adolescents with LIQ reported being more angry (*M* = 2.21, *SD* = .91) than BIQ (*M* = 1.76, *SD* = .51) and AIQ groups (*M* = 1.60, *SD* = .48; *F*(2,216) = 14.70, *p* < .001, *η*^2^ = .12, see Fig. 1). BIQ and AIQ adolescents did not differ significantly from one another. No differences in Anger were observed between the IQ groups in the hostile situations nor the accidental situations. No effects were found for Externalizing Behavior.

#### Self-Efficacy for Aggressive Responses

No significant interaction effects were found for Self-efficacy for Aggressive Responses; however, three main effects indicated pervasive differences for this SIP skill (Tables 3, 4, 5). The main effect for Situation Type revealed that when the intent of the perpetrator was clearly hostile, adolescents across all groups had the highest self-efficacy for being aggressive, compared to ambiguous and accidental situations. The main effect for Cognitive Level revealed that adolescents with LIQ had higher self-efficacy for aggressive responses than the adolescents in the AIQ group, but the LIQ and AIQ groups did not differ significantly from the BIQ group. The main effect for Externalizing Behavior revealed that the behavior problems group had higher self-efficacy for being aggressive compared to adolescents in the non-clinical group. These main effect analyses revealed that the SIP skill of Self-Efficacy for Aggressive Responses was pervasively elevated in adolescents with LIQ and with behavior problems, and for all adolescents in general in hostile situations specifically.

#### Evaluation of Aggressive Responses

A significant two-way interaction of Situation Type by Cognitive Level was found for Evaluation of Aggressive Responses (Pillai’s Trace *V* = .07, *F*(4,424) = 3.93, *p* = .004, *η*^2^ = .04), without effects for the covariates. Post-hoc probing tests revealed that, in ambiguous situations, adolescents with LIQ evaluated aggressive responses more positively (*M* = 1.60, *SD* = .69) than adolescents in the AIQ group (*M* = 1.38, *SD* = .49; *F*(2,216) = 3.95, *p =* .02, *η*^2^ = .04), but not more than adolescents in the BIQ group (*M* = 1.53, *SD* = .66; see Fig. 1). The AIQ and BIQ groups did not differ significantly from one another in the ambiguous situations. No group differences were found in the hostile situations nor the accidental situations. No effects were found for Externalizing Behavior.

#### Selection of Aggressive Responses

A significant two-way interaction of Situation Type by Cognitive Level was found for the Selection of Aggressive Responses (Pillai’s Trace *V* = .05, *F*(4,424) = 2.88, *p* = .02, *η*^2^ = .03), without effects for the covariates. Post-hoc probing tests revealed that, in ambiguous situations, adolescents with LIQ were significantly more likely to select an aggressive response (*M* = .35, *SD* = .64) than adolescents in the AIQ group (*M* = .01, *SD* = .12; *F*(2,216) = 9.81, *p* < .001, *η*^2^ = .08), but not compared to the BIQ group (*M* = .18, *SD* = .43; see Fig. 1). The AIQ and BIQ groups did not differ significantly from one another in the ambiguous situations condition. Visual inspection of the means in Fig. 1 suggested that adolescents in the LIQ group may also be more likely to select aggressive responses in the hostile situations compared to adolescents with BIQ or AIQ; however, these differences were nonsignificant for Cognitive Level (*F*(2,216) = 2.70, *p* = .07, *η*^2^ = .02). In accidental situations, there were no significant differences between the IQ groups for the selection of aggression.

In the repeated measures ANCOVA, the interaction effect between Situation Type and Externalizing Behavior for Selection of Aggressive Responses was nonsignificant (Pillai’s Trace *V* = .03, *F*(2,211) = 2.84, *p* = .06, *η*^2^ = .03), in contrast with the significant outcome from the overall MANCOVA (*p* = .045, see Table 2), which is explained by variance differences as this multivariate analysis took into account the full SIP model. Consistent with the results of this overall MANCOVA analysis, post-hoc probing tests revealed that, in accidental situations, adolescents with behavior problems selected significantly more aggressive responses (*M* = .31, *SD* = .57) than non-clinical adolescents (*M* = .15, *SD* = .39; *F*(1,216) = 9.52, *p* = .002, *η*^2^ = .04; see Fig. 2). In addition, in hostile situations, the behavior problems group also selected significantly more aggressive responses (*M* = .52, *SD* = .69) than the non-clinical group (*M* = .26, *SD* = .57; *F*(1,216) = 8.58, *p* = .004, *η*^2^ = .04). In the ambiguous situation condition, there was no difference in selection of aggressive responses between the behavior problems groups.

## Discussion

In the current study we found evidence for situation-specific SIP in ambiguous, but also clearly in accidental situations: four SIP skills were biased in adolescents with low intellectual level, congruent with a mild intellectual disability (interpretation of purposeful intent, related feelings of anger, and evaluation and selection of aggressive responses), both in accidental and ambiguous situations. Two SIP skills were biased in adolescents with behavior problems (aggressive response generation and selection), both in accidental and hostile situations.

### Interpretation Skills Differ in Accidental Situations in Adolescents with Low Intellectual Level

We provided insight into the full scope of SIP deficits or biases from the early, middle and late phases of the SIP model. In line with Leffert and Siperstein (1996) we first anticipated that adolescents with low intellectual level (LIQ), congruent with mild intellectual disability, would attribute hostile and purposeful intent to perpetrators in all social situations, and specifically more than their typically developing peers in situations with accidental or ambiguous intent. We found a pervasively biased interpretation of hostile intent for adolescents with LIQ in all situation types, indicating a prominent but not situation-specific interpretative hostile bias for adolescents with low intellectual level, in line with Van Nieuwenhuijzen et al. (2004).

Moreover, adolescents with LIQ more often attributed purposeful intent to perpetrators than peers did with BIQ or AIQ, and as expected, specifically more so in accidental situations. Though the medium effect size should be interpreted cautiously, these findings are consistent with results reported by Leffert and Siperstein (1996) on inaccurate interpretations of benign intentions by younger children with ID. The findings are also consistent with previous research showing that youth with ID have maladaptive SIP skills, especially when the information in the social situation is more complex (Van Nieuwenhuijzen et al. 2011). The interpretation bias in accidental situations was not found for youth with behavior problems. The outcome may be mostly explained by the amount of variance explained by the general cognitive level factor, accounting for more variance than the behavior factor. Attributing an accidental or benign nature to a perpetrator’s intent in a situation where the victim experiences a negative outcome may produce a higher cognitive load (Rosset and Rottman 2014). Youth with LIQ may fail to integrate multiple contradicting pieces of information about the perpetrator, context, and outcome into one benign conclusion. Such vulnerability in the LIQ group for having a biased negative interpretation can lead to a negative cycle of SIP and aggressive behavior (Dodge et al. 2015), and presents a challenge to clinical work treating youth with LIQ on their social and behavior problems.

### Situation-Specific Differences in the Full Scope of SIP Skills

We also hypothesized that accidental situations as well as ambiguous situations would elicit differences between groups on the later SIP skills of generation, evaluation, self-efficacy, and selection of aggressive responses and related feelings of anger. Confirmation was found for biased SIP skills of evaluation and selection of aggressive responses and related feelings of anger in adolescents with LIQ in ambiguous situations, however, not in the accidental situations. These findings for adolescents were thus consistent with the seminal SIP studies (Dodge 1980; Dodge et al. 1984) and previous studies about SIP differences between children with ID and typically developing peers in ambiguous situations only (e.g., Van Nieuwenhuijzen et al. 2004, 2011). The ambiguous situations may produce complexity for processing, leading youth to fall back to familiar cognitive and behavioral options that may limit youth with LIQ in evaluating and selecting optimal solutions in new social situations (Rosset and Rottman 2014).

Additionally, although adolescents with behavior problems did not show interpretation biases in accidental situations specifically, they generated and selected more aggressive responses in these situations than non-clinical adolescents, regardless of IQ. Thus, independent of the other’s intention, these youth generated an aggressive solution possibly because they felt disadvantaged anyway. Social problem situations, even when accidental, may be complex for adolescents with behavior problems because they may provide an unsafe state to which they respond with anger or selecting aggressive responses as the best option from presented alternatives. As found by Calvete and Orue (2012), previous negative experiences or cognitive schemas about hostility may arise which make these youth hold on to generating and selecting well-known responses. These responses have been evaluated successfully, even if these were aggressive. The adolescents with behavior problems were also more likely than their peers to select aggressive responses from several presented options in situations involving hostile intent, which may lead to escalating hostility in real-life settings. These situation-specific results were not found as a function of cognitive level, but importantly, independent of their level adolescents with behavior problems show SIP deficits and biases that are characteristic for this clinical group, even in accidental situations. Therefore, these findings once again underscore the value of examining SIP across a range of social situations, not only in ambiguous situations (Dodge et al. 1984; Matthys et al. 2001; Nas et al. 2005).

It is notable that both adolescents with externalizing behavior problems and adolescents with LIQ were more likely to select aggressive responses than their peers, although their aggressive choices were observed in different types of situations. This SIP cognition is the final cognitive step in the SIP model before an actual behavior is enacted and it has not received as much attention in the literature as other SIP skills, such as interpretation (but see Fontaine et al. 2002; Matthys et al. 1999; Van Nieuwenhuijzen et al. 2005). It is remarkable that even after adolescents had completed a full assessment of their decision-making including extensive questions and evaluations of a variety of nonaggressive response options, adolescents in these groups were still more likely to select aggressive responses. One might expect that simply the process of considering a series of questions within the assessment itself could positively influence youth and change their views on selecting “adequate”, adaptive or prosocial, responses in the end (Lemerise et al. 2005). In spite of this assessment process, the fact that aggressive patterns of responding emerged suggest that more research on determinants of response selection is needed.

The externalizing behavior factor was thus strongly associated with biases in generating and selecting aggression, comparable with the studies by Dodge and colleagues (Dodge 1980; Dodge et al. 1984; Crick and Dodge 1994), whereas for most other SIP deficits the cognitive level factor was most influential, in line with Leffert and Siperstein (1996), and Van Nieuwenhuijzen and colleagues (2006; Van Nieuwenhuijzen et al. 2009b; Van Nieuwenhuijzen et al. 2011). Somewhat surprisingly, behavior problem group differences in interpretation were not found in ambiguous situations. These findings are in contrast with previously reported differences in SIP deficits examined in ambiguous situations (e.g., Kupersmidt et al. 2011). This difference may be explained by inclusion of the factors externalizing behavior and cognitive level simultaneously. As can be seen by the effects sizes in the tables, for most SIP skills cognitive level may have accounted for such strong effects that differences for externalizing behavior were no longer significant.

### Pervasive SIP Deficits and Biases for Encoding and Self-Efficacy

In contrast with our hypothesis, biases on the SIP skill of self-efficacy for aggression were not found to be situation-specific; however, three main effects were found. The highest self-efficacy scores for aggression were found for adolescents with ID, for adolescents with behavior problems, and in general in hostile situations, but not in significant interaction with one another. As the three main effects showed medium to (very) large effect sizes, these differences as a function of cognitive level, externalizing behavior, and hostile situations in general should be taken into account when clinically assessing self-efficacy in an individual adolescent for a better understanding of the vulnerability for maladaptive social behavior.

Finally, for the cognitive encoding skill, differences between groups were expected to be more pervasive, and less situation-specific. Confirmation was found for this hypothesis, meaning that adolescents with ID are pervasively impaired in encoding information, regardless of the social problem situation these adolescents find themselves in. This supports the idea that encoding is a more purely cognitive skill, and thus linked with the general cognitive impairment of youth with ID.

### Limitations and Recommendations

The outcomes of the current study should be placed in perspective of its limitations. First, SIP skills were created as mean or sum scores based upon responses to questions about two video recorded vignettes per situation type; a method different from studies that have utilized a larger number of videos or scenarios, such as Kupersmidt et al. (2011) but that only used videos of ambiguous situations. The decision for using a smaller number of videos per situation condition was made to produce feasible test durations and valid outcomes for youth with ID, who would not tolerate lengthier test administrations. It may have limited the reliable measurement of individual SIP skills; however, our hypothesized situation-specific results support that differences between the situations can be validly tested by two videos per type. Second, the two videos for each condition consisted of one provocation by a peer and one provocation by an adult perpetrator, as these were reported by adolescents themselves as common and valid problematic situations (Van Bokhoven et al. 2011). The inclusion of adult perpetrators may have contributed to the variability in responding within an individual SIP skill between the two types of videos per condition. When studying more similar (peer) situations, as in previous literature, it is thus expected that current situation-specific results would become even stronger.

Third, due to the large amount of variables within the repeated measures MANCOVA (3 situation types by 3 IQ groups by 2 behavior groups on 8 SIP outcomes with 2 covariates), false discoveries may be made. However, it must be noted that we controlled for Type 1 error by omnibus testing in one repeated measures MANCOVA. Moreover, only in case of multivariate significant results from the MANCOVA the next specifying steps were performed by investigating univariate results, subsequent ANCOVAs and post hoc tests, confirming these multivariate results. Even more so, our (very) strong and significant multivariate results (including eight SIP skills) provide a strong support for how robust SIP differences are between groups and between situations.

Fourth, we divided participants into groups of cognitive level based primarily upon their IQ scores. According to the definition of intellectual disability, however, (social) adaptive functioning problems should be taken into account as well when classifying individuals with an intellectual disability (Schalock et al. 2010). We supported our grouping procedure based on the IQ-estimate scores, because of our recruitment from specialized institutions for the care of youth with mild ID and borderline intellectual functioning. As we did not have strong a priori hypotheses for the BIQ group, our findings about SIP in the BIQ group should be interpreted with caution. We propose a more thorough investigation of SIP skills, including the intellectual and (social) adaptive functioning problems in youth with borderline intellectual functioning (Peltopuro et al. 2014).

### Clinical Implications

Social cognitive processes are targets of cognitive behavior therapy. Children learn to adequately interpret the intentions of others and subsequently generate appropriate non-aggressive responses. For example, in the Coping Power program (Lochman et al. 2008) children learn to label the intention of others into one of four categories (accidental, prosocial, hostile, ambiguous) and subsequently generate and evaluate various responses. Findings of the present study support the need of including problem situations that are clearly accidental in nature and are still difficult for adolescents with low IQ, congruent with mild ID, to interpret adequately. For both adolescents with clinical levels of externalizing behavior and for adolescents with low IQ findings support the need of working on the evaluation and selection steps in cognitive behavior therapy.

## Conclusion

Current findings show that the type of social problem situation reveals situation-specific biases and deficits in several SIP skills, especially for youth with low IQ, congruent with mild ID, and youth with clinical levels of externalizing behavior. SIP deficits and biases were not only brought out by ambiguous situations, but by accidental situations as well. These findings add to the existing literature on SIP deficits and biases found for children with ID and with behavior problems in ambiguous situations. Our results suggest that in the assessment and understanding of SIP, especially in clinical groups of youth, more attention should be paid to situations in which youth are clearly accidentally disadvantaged.
